# Barriers and facilitators to participant recruitment and retention among black adults in a mobile health intervention to control hypertension (MI-BP): A mixed methods study

**DOI:** 10.1017/cts.2026.10738

**Published:** 2026-05-06

**Authors:** Analay Perez, Katee Dawood, Reema Kadri, Rachelle Muladore, Alexandria Degner, Dongru Chen, Dara Harris, Phillip Levy, Lorraine R. Buis, Timothy C. Guetterman

**Affiliations:** 1 Department of Family Medicine, University of Michigan, Ann Arbor, USA; 2 Department of Emergency Medicine and Integrative Biosciences Center, Wayne State University, Detroit, USA

**Keywords:** Recruitment, retention, mHealth, hypertension, blood pressure

## Abstract

**Introduction:**

There are growing efforts to recruit and retain individuals from various populations in clinical trials to increase trial representativeness. Nonetheless, these challenges can hamper the development of clinical trials, contributing to increased inequities. This study explored the barriers and facilitators of participating in a mobile health trial designed to improve blood pressure (BP) among Blacks with uncontrolled hypertension from underserved communities.

**Methods:**

Participants were recruited from a larger mHealth clinical trial, MI-BP, across emergency departments, mobile health units, and community-based settings. We conducted an explanatory sequential mixed methods design to quantitatively examine participants’ experiences with the MI-BP trial including satisfaction and reasons for dropping out. The qualitative semi-structured interviews expanded on participant experiences based on the quantitative results. Quantitative and qualitative results were integrated to provide a more comprehensive understanding.

**Results:**

Fifty-two participants completed the survey and a subset of 22 were interviewed. There were no statistically significant differences on reasons for joining the MI-BP study regardless of study completion. Participants were generally motivated to learn about ways to improve their BP, with many noting positive experiences, including completers and non-completers. Some who dropped out indicated meeting their goal of lowering their BP. Despite a robust consent process, some in the non-completer group reported not understanding certain components of the study compared to the completer group. Additional barriers included concerns over adverse effects and missing worktime.

**Conclusion:**

Findings illuminate the barriers and facilitators participants encountered in the MI-BP trial and provides considerations for reducing barriers among this population.

## Introduction

Recruiting and retaining participants in clinical trials can be complex and challenging due to participant demographics, personal attributes, study concerns (e.g., intervention side effects), and lack of support from the research team [1,2]. Clinical trials commonly experience delays, compounded by difficulties recruiting and retaining individuals, leading to increased study costs and protracted timelines [1]. Recruitment is hindered by participant burden, such as requesting time off from work to participate, traveling to the study site, and experiencing actual or perceived adverse events from the intervention [2]. Beyond recruitment, participant retention can also critically influence study outcomes. Family and caregiving responsibilities, lack of transportation, and fear or emotional distress from intervention activities like major behavioral changes further complicate retention [3,4].

Addressing participant recruitment and retention issues in clinical trials is vital for reducing attrition and ensuring the sample accurately represents the focal population [5,6]. Representativeness is crucial when working with historically marginalized populations. Including diverse populations in clinical trials allows researchers to better understand differences in intervention uptake across populations and informs intervention adaptations to reduce health inequities. The US Black population is one of the groups most significantly affected by inequities and is often challenging to recruit and retain for clinical studies [7].

The success of a clinical trial relies heavily on participants’ willingness to volunteer and the strategies researchers implement to retain participants throughout the study. With participant recruitment and retention already being a challenge, power differentials with researchers and mistrust in healthcare [7] present further barriers to recruiting Black patients. Participant retention is equally crucial, as retention is a continuous process that affects the reliability and validity of results [8,9]. Effective strategies are needed to maximize participant recruitment and retention rates in clinical trials involving Black populations and enhance their representation in clinical trials [10].

Recruitment and retention of diverse groups are even greater concerns when seeking to reduce existing inequities. According to the National Health and Nutrition Examination Survey (NHANES), Black Americans have a higher prevalence rate of hypertension (45.3%) and experience about five times the mortality rate from hypertension compared to White Americans [11] To conduct clinical trials focused on health equity, the ability to recruit and retain diverse populations, especially Black patients, is crucial.

The MI-BP intervention consisted of a mobile health (mHealth) app that promoted home BP monitoring, physical activity tracking with a Fitbit pedometer, dietary sodium tracking within the app, and medication adherence reminders. The intervention included goal setting and tailored messaging to affect behavioral factors associated with blood pressure (BP) control. Participants in the MI-BP intervention were given a BP cuff and were then randomized across the following arms: (a) either enhanced usual care control (usual care + home BP monitoring) or (b) the MI-BP mHealth intervention for one year [12,13]. Although mHealth initiatives aim to reduce treatment barriers in Black populations, recruitment and retention was an obstacle in the MI-BP trial [13]. Therefore, this study aimed to investigate the reasons for high loss to follow-up rates before participant randomization and high dropout rates during a 12-month randomized controlled mHealth trial to improve BP among Black individuals with uncontrolled hypertension. This study was based on the Perceptions and Insight study that examines participation in clinical research [14]. To investigate the barriers and facilitators to participant recruitment and retention among Black individuals in clinical studies, mixed methods approaches can help quantify variables influencing participants’ decisions to complete or drop out of the trial and explore their firsthand experiences. Therefore, using a mixed methods approach allowed us to examine the factors that contributed to recruitment and retention of study participants in the MI-BP trial, and using the quantitative results, we explored and gained additional nuance and perspectives across individuals of varying study participation about their experiences in the trial pertaining to their engagement. A mixed methods study consisting of surveys and semi-structured interviews was conducted among participants who completed the entire trial and those who were partially engaged or did not complete the MI-BP trial. This study was guided by the following research questions:

1. What factors influenced participant recruitment and retention in the MI-BP trial among completers and non-completers? (Quantitative)
2. How did participants describe their motivations and demotivations to participate and engage in the MI-BP trial? (Qualitative)
3. What barriers and facilitators to participant recruitment and retention did participants experience across completers and non-completers? (Mixed Methods)

## Materials and methods

### Design

This study stemmed from a larger mHealth clinical trial [12,13,15]. For the current study, we employed an explanatory sequential mixed methods design, which involved collecting and analyzing quantitative data first, followed by qualitative data collection and analysis to help explain the quantitative results [16]. The quantitative phase consisted of a brief survey examining participants’ experiences with the MI-BP trial. Results from the quantitative phase informed the sampling approach for the follow-up qualitative phase. The qualitative phase consisted of a qualitative descriptive study including individual semi-structured interviews with participants who completed the MI-BP trial, who partially completed the MI-BP trial, and those who did not complete any portion of the MI-BP trial after screening and consent. The qualitative descriptive design allowed us to generate in-depth understanding and themes about participants’ engagement or disengagement in the trial and better explain the quantitative findings [17]. This study followed the Good Reporting of a Mixed Methods Study (GRAMMS) guidelines [18] (Supplementary Material 1).

### Participant recruitment and eligibility

Participants in the parent trial [12,13] were recruited from one of two Detroit based emergency departments, mobile health units, and community-based settings. To be eligible to participate, participants were required to be Black individuals, between the ages of 25–70 years old, with a self-reported history or diagnosis of hypertension, owned a smartphone, and presented with uncontrolled BP (systolic BP ≥ 135 mm Hg) at triage/meeting and on repeat measurement using the BpTRU BPM-200 device (Smiths Medical PM, Inc.) or Omron HEM 907XL IntelliSense (Omron Healthcare, Inc.). Participants in the parent trial could receive up to $275 over the course of one year for their participation in the study. To increase retention efforts in the parent trial, transportation was provided for those who needed it for study visits via taxi or ride-sharing services. The study protocol [12] and trial outcomes [13] have been published elsewhere. For this study, participants who consented from the larger trial were invited via a text message with a REDCap link to complete the survey. To be eligible for this study, we recruited participants who had completed all components of the MI-BP study and those who partially engaged (completed at least one component of the study) or were consented but did not participate in any component of the study.

### Ethical considerations

Institutional review boards approved the study at two Midwestern institutions (HUM00114202 and IRB 040416M1F). Participants provided informed consent before enrolling in the study.

### Quantitative surveys

A brief survey, adapted from the Clinical Research Participation Perceptions and Insights Survey, was used to assess participants’ motivations (or demotivations) for participating in the study, their experiences and satisfaction with the MI-BP trial, and their satisfaction with the MI-BP staff [14]. For participants who did not complete any intervention component, the survey questions focused on their motivations to participate, experiences with the MI-BP study, and reasons for dropping out. The survey consisted of 7–10 questions (some questions were skipped based on participant response) and responses ranged from *very important* to *not important at all*, with higher scores representing increased positive endorsement (e.g., the consent form was easy to understand). The survey was categorized into two major sections, questions regarding their desire to join the study (i.e., “Why did you join?”) and experiences with MI-BP. The survey questions regarding participants desire to join the study had an acceptable internal consistency (Cronbach’s alpha = 0.74). The survey questions centered on participants’ experiences with MI-BP demonstrated an excellent internal consistency (Cronbach’s alpha = 0.96). For analysis, we categorized participants into two groups – (1) those who completed all components and visits of the study (i.e., completers) and (2) those who partially engaged or did not complete any portion of the research study (i.e., non-completers). We believed the reasons for *some* engagement and no completion would be most similar.

We aimed to examine differences between completers and non-completers across three primary areas: reasons for joining, experiences with the MI-BP research study, and reasons for dropping out among non-completers. Participants who completed the survey received a $20 ClinCard that functioned like a debit card. To encourage response rates, participants who submitted the survey within the first, second, or third week of release were entered into a lottery, and up to 40 participants were eligible to receive monetary incentives of up to $100. Quantitative analysis using SAS (version 9.4; SAS Institute) included Wilcoxon rank-sum test or Fisher’s Exact test, as appropriate, to examine group differences and descriptive statistics. Participants completed this survey online via REDCap and took an average of seven minutes to complete (Survey instrument is provided in Supplementary Material 2).

### Qualitative interviews

Upon completion of the surveys, participants who indicated interest in the follow-up phase were invited to participate in a semi-structured interview using a connecting integration technique [16]. All interviews were conducted via Zoom and lasted approximately 15–30 minutes. These interviews aimed to comprehensively explore the barriers and facilitators to participation in the MI-BP study. We developed interview questions that expanded on concepts addressed in the survey to prepare for integrating quantitative and qualitative results through a matching process. Participants were asked several questions, including their initial experience with the MI-BP study, level of engagement, perceptions of study visits, concerns regarding confidentiality and privacy, and reasons for stopping the study. (Interview guide is provided in Supplementary Material 3). The incentive amount for the interview ranged according to their study involvement due to the expected length of the interview (i.e., participants who completed the trial and those who partially engaged were offered a $100 ClinCard, and those who did not participate to any degree were offered a $50 ClinCard).

All interviews were audio-recorded, professionally transcribed, and imported into MAXQDA 2024 for qualitative analysis [19]. To analyze the semi-structured interviews, we employed a thematic text analysis process that involved iterative coding of transcripts using open coding. After open coding to develop a preliminary codebook, two researchers on the team met to discuss the codebook and independently coded two transcripts. After coding discussions, they independently coded the remainder of the transcripts and met regularly to discuss questions/discrepancies. Then, the analysts examined codes for patterns and commonalities to develop topical categories and subcategories by grouping similar codes and then combined similar categories to generate themes [20]. We conducted interviews until thematic saturation was met, where no additional complexity was added to the themes. To ensure the trustworthiness of the findings, the two analysts held a peer debriefing session to discuss the findings of the study with the larger study team and make any modifications to clarify or expand themes. Through an integrative mixed methods analysis, the quantitative and qualitative results were merged to explain further the initial quantitative results [21]. We aligned the quantitative and qualitative results based on common domains (e.g., reasons for joining the MI-BP trial, study experiences with MI-BP trial, and reasons for leaving the study). We used the qualitative results to expand and further explain the quantitative results by comparing results between completers and non-completers. We engaged in this process iteratively to develop meta-inferences using visual joint displays [22].

## Results

### Participants

Fifty-two participants completed the survey, 22 of whom (42%) completed the MI-BP trial (i.e., completers). The other 30 (58%) completed some portion (*n* = 10) or did not engage in the trial (*n* = 20) (i.e., non-completers). Most participants were female (*n* = 42, 81%) and ten (19%) were male. Table 1 displays demographic data.

**Table 1.** Sample demographic Table 1 long description.

Characteristics	N (%)
***Education***	
Lower-than-college education	14 (35.9%)
Some college education	11 (28.2%)
College/technical degree	14 (35.9%)
***Insurance***	
Medicaid	15 (38.5%)
Private health insurance	11 (28.2%)
Medicare	7 (18%)
Uninsured	6 (15.4%)
***Employment***	
Employed	26 (66.7%)
Other	13 (33.3%)
***Annual Income***	
Less than $10,000	12 (30.8%)
$10,000–$24,999	5 (12.8%)
$25,000–49,999	9 (23.1%)
$50,000	4 (10.3%)
Did not report	9 (23.1%)

*Note:* Demographic data are available for 39 participants.

### Quantitative

Both groups were asked about their reasons for joining the MI-BP study. Reasons for joining the MI-BP study did not significantly differ across completers and non-completers based on the Wilcoxon rank-sum tests or Fisher’s Exact tests (Table 2). We used median rather than the mean because it is a more robust measure for ordinal Likert-type scales [23]. Although not statistically significant, the only median scores that differed between completers and non-completers was joining the study due to monetary compensation (*Mdn*_completer_ = 3.00, *Mdn*_non-completer_ = 4.00).

**Table 2.** Comparisons of “Why did you join?” between completers and non-completers Table 2 long description.

Why did you join?	Completers (*N* = 22)	Non-completers (*N* = 30)	P-value*
	Median	Median	
Help advance science and the treatment of high BP	5.0	5.0	.57
Help save or improve the lives of other patients	5.0	5.0	.07
Help improve my own high BP	5.0	5.0	.99
Represent the best treatment option	5.0	5.0	.99
The monetary compensation provided for participation	3.0	4.0	.98
Guide understanding of how well a new BP program works	5.0	5.0	.32
Receive more care and attention from health professionals	5.0	5.0	.22
Give me something to do	3.0	3.0	.75
Give me someone to talk with	3.0	3.0	.87
*Comparisons of experiences with MI-BP between completers and non-completers*			

Experiences with MI-BP	Completers (*N* = 22)	Non-completers (*N* = 30)	P-value*
	Median	Median	
The consent form was easy to understand	5.0	3.5	**.04**
The study fit well into to my daily routine	4.0	3.0	.07
The number of study visits was just right	4.0	3.0	.07
The length of study visits was just right	4.0	3.0	**.02**
The overall amount of time the study took was about right	4.5	5.0	.79
Receiving compensation was convenient	4.0	4.0	.88
The compensation was enough	4.0	4.5	.93

*Note:* *Comparisons between completers and non-completers were calculated using Wilcoxon rank-sum tests or Fisher’s Exact tests, as appropriate; a higher median value indicates more agreement.

We also examined group differences between completers and non-completers regarding their experiences with the MI-BP study. We found a statistically significant difference between groups in understanding the consent form and the length of study visits. Participants who completed the trial found the consent form easier to understand (*Mdn* = 5.00) than non-completers (*Mdn* = 3.50) (*p* = .04). Participants in the completer group also found the length of study visits appropriate (*Mdn* = 4.00) at a significantly higher rate than non-completers (*Mdn* = 3.00, *p* = .02) (Table 2).

Participants rated their level of satisfaction on a 5-point Likert scale ranging from 1 (very dissatisfied) to 5 (very satisfied). There were no significant differences in participant satisfaction on the helpfulness of MI-BP study staff in aiding participants’ decision-making about participation between those who completed the trial (*Mdn* = 5.00) and those who completed only some components (*Mdn* = 5.00, *p* = .50) or helpfulness answering questions between both groups (*Mdn* = 5.00, respectively, *p* = .32). On average, participants’ reasons for dropping out of the study included possible risks to their overall health (Mean = 3.89, SD = 1.08), concerns about their private medical information becoming public (Mean = 3.72, SD = 1.13), possibility of missing too much work time (Mean = 3.59, SD = 1.42), and potential adverse effects (Mean = 3.56, SD = 1.15) (Table 2).

### Qualitative

Twenty-two participants were interviewed, including 15 who completed the trial and seven non-completers. Three themes were identified: *motivations to learn about BP enhance recruitment, helping to monitor and maintain BP keeps participants engaged*, and *facilitators to recruitment and retention with proposed actionable next steps.*

### Motivations to learn about BP enhance recruitment

Participants across both the completer and non-completer groups discussed several motivations for participating in the MI-BP study, primarily driven by their desire to learn more about their health and well-being (Table 3). These included learning how to control their BP, identifying BP treatments across different groups, learning about factors influencing BP (e.g., family history, age, health), and receiving information on accessing medication. These motivations were facilitators to study engagement. Some participant’s primary goal for joining the study was to access prescriptions for medications they were unable to acquire. Participants reported that their BP medication was not effective prior to the MI-BP trial, but through study participation, some found the appropriate prescriptions through the study. Some participants were able to receive medication at no cost, as our staff helped facilitate connections to free and low-cost medications and resources for those who were eligible. As such, this also served as a facilitator to study participation and engagement.

**Table 3.** Motivations to learn about BP enhance recruitment theme and subthemes Table 3 long description.

Subthemes	Summary	Exemplar quotes
Learning to improve health (facilitator)	Participants described being motivated to participate in the study for personal health-related reasons such as improving their overall health.	“…just a longing to want to have my BP controlled. My current physician didn’t seem to be having any success with managing my BP.” (P5; completer) “Because I know I had very bad high BP and they would really show me how to control it and keep it maintained. Because I was having severe headaches.” (P9; non-completer)
External motivators (facilitator)	Participants discussed being motivated to participate in the study due to external stimuli such as a reward or incentive.	“I liked received my BP machine, I liked that, and I had liked the Fitbit watch, I liked that. And it was helping me take my medicine and keeping it on track.” (P15; completer)

Participants across both groups also expressed their desire to learn from the research. Participants in the completer group sought to understand the meaning and implications of systolic and diastolic BP levels and how factors such as diet, weight, stress, and genetics could influence high BP. Participants in the completer group also reported a strong motivation to learn about BP treatment among Black communities and variations in medication efficacy across these populations, serving as a facilitator.

The completer group predominantly reported external motivators compared to the non-completer group, including access to healthcare through either medication or professional support due to a lack of medical insurance, study staff openness, receiving a blood cuff monitor/Fitbit, and study site convenience within their community. Only one participant from the non-completer group reported an external motivator – the desire to try something new by participating in research.

### Helping to monitor and maintain BP keeps participants engaged

The primary reason for engaging in the study among both groups was that it enabled participants to monitor and maintain healthy BP levels through prescriptions for medications (Table 4). Additional facilitators included a personal desire to follow through with the study, transportation and monetary incentives, and friendly study staff. Participants also shared several factors that increased their participation such as the desire to feel good overall, learning about BP, medical testing, and noticing improvements in BP.

**Table 4.** Helping to monitor and maintain BP keeps participants engaged theme and subthemes Table 4 long description.

Subthemes	Summary	Exemplar quotes
Reasons for continuing study (facilitator)	Participants discussed reasons for continued engagement in the study including monitoring BP, especially through medication, desire to follow-through, and several other reasons.	“Because I appreciated the help, because I was getting like medications for my hypertension and you guys helped provided that for me.” (P4; completer)
Reasons for stopping study (barrier)	Participants noted reasons for dropping out of the study including achieving the goal of lowering BP, feeling better, and one participant did not remember reasons why they stopped the study.	“They [research staff] achieved the successful mission of bringing down my BP. Now in the event that it did rise up after I had stopped going, it was because of stressful situations that was out of my hands.” (P19; non-completer)
Influence of COVID-19 on study participation	Participants expressed changes to their study participation in relation to the COVID-19 pandemic and how this influenced their participation in the study.	“I thought the program might have disappeared or something, I didn’t try to reach in my notes and try to find out who I could call to find out about you guys. But it wasn’t me trying to stop going to the program, it was just that it looked like the program stopped after the pandemic situation from my perspective.” (P9; non-completer)

Seven participants from the non-completer group shared barriers to study participation and engagement, including feeling ill from changing medications or achieving their study goal of lowering and controlling BP. Most participants reported no disruptions to the study before the COVID-19 pandemic or changes in participation following the pandemic. However, most appointments switched from in-person to over-the-phone. One participant mentioned experiencing limited communication with the study team, and two participants from the non-completer group reported not receiving follow-up information during COVID.

### Facilitators to recruitment and retention with proposed actionable next steps

Eight participants recommended study modifications to increase retention efforts (Table 5). Three participants from the completer group and two from the non-completer group reported the need to reduce the length of surveys because they required a significant amount of time to complete. Not all participants wanted the devices offered through the study and instead preferred to choose a device. For example, one participant wanted to choose the device they received (e.g., BP cuff or Fitbit and BP cuff), and another participant recommended hosting seminars to shed light on participants’ experiences. One participant from the non-completer group suggested an increase in monetary incentives.

**Table 5.** Facilitators to recruitment and retention with proposed actionable next steps Table 5 long description.

Consideration	Summary	Exemplar quotes	Actionable next steps
Length of surveys (barrier)	Participants discussed the length and repetitiveness of the surveys as a barrier.	“The part of the program I didn’t like too well, it was too many questions to answer on that tablet. And I’m not computer savvy.” (P9; non-completer)	Review surveys for redundant questions and aim to shorten surveys (if possible) to lower completion time.
Offer increased monetary incentives (facilitator)	Participant discussed and invited increased monetary incentives for study participation.	“I think unless you want to offer more money. But other than that, I think it was fine as it was. And I stayed in it for as long as y’all stayed in touch with me.” (P14; non-completer)	Consider if and how monetary incentives could be increased or identify alterative retention strategies regarding incentives that could be tailored to the population.
Hosting community seminars (facilitator)	One participant was very vocal about the value of hosting community seminars about people’s experiences with BP management.	“…maybe talking at seminars, sometimes when people are more receptive when they don’t see a lot of degrees and we sharing your experiences with other people, as opposed to people getting up – I’m not taking away from people with degrees or whatnot, but sometimes you being on the same level as just being a Black woman or just being a woman or just or just being a person with high BP, sometimes people will receive that message a little bit more than a bunch of professors and doctors or whatnot getting up there talking.” (P21; completer)	Inviting participants to speak about high BP at community events to help others and increase receptivity to seeking treatment.

### Integrated results

Table 6 presents the joint display, a visual depiction of integrated quantitative and qualitative results, and mixed methods meta-inferences [22]. Participants’ reasons for joining the MI-BP study did not differ between completers and non-completers. Participants in both groups noted several motivating factors for participating in the study, largely influenced by their motivation to learn how to improve their BP. Others shared external motivating factors such as receiving a blood cuff monitor or Fitbit and the study staff’s openness when interacting with them.

**Table 6.** Joint display of integrated quantitative and qualitative findings with corresponding meta-inferences Table 6 long description.

Quantitative research aims	Quantitative summary results	Qualitative themes and summary results	Meta-inferences
Reasons for joining the MI-BP trial study	No statistically significant differences between completers and non-completers regarding reasons for joining. Median values remaining consistent on all items across both groups. Only one item demonstrated a difference in median scores regarding whether participants were influenced by monetary compensation but was not statistically significant (*Mdn*_completer_ = 3.00, *Mdn*_non-completer_ = 4.00, *p* = .98).	*Motivations to learn about BP enhance recruitment learning to improve health* Learning to improve health included learning about BP and ways to control BP. *External motivators* External motivators included receiving access to healthcare or doctor visits, receiving a BP cuff/Fitbit, and the convenience of the research center.	Motivation for participating in the study did not differ across completers and non-completers. Participants in both groups reported similar motivating factors such as access to healthcare and BP cuff/Fitbit as well as learning about ways to control BP. Both groups were motivated by tangible methods of improving their BP and overall health, regardless of completion or non-completion of trial.
Experiences with MI-BP trial	Across items measuring participant experiences with the MI-BP trial, two items were found to be statistically significant between completers and non-completers – understanding the consent form and length of study visits. In both cases, completers found the consent form easy to understand at a higher level (*Mdn* = 5.00, *p* = 0.04) than non-completers (*Mdn* = 3.5) and found the length of study just right (*Mdn* = 4.00, *p* = 0.02) in comparison to non-completers (*Mdn* = 3.00). There were no statistically significant differences between completers and non-completers on average satisfaction with MI-BP study staff.	*Helping to monitor and maintain BP Keeps participants engaged* Main reason for high retention rates across both groups was to monitor and maintain healthy BP levels, including their access to medication. Facilitators such as providing transportation and monetary incentives, friendly study staff, and desire to improve overall health kept participants engaged in the study. Some dropped out of study because they met their goal of lowering their BP or because the medication made them feel ill.	Both groups experienced a positive experience with the MI-BP trial and reasons for these were expanded through the qualitative findings, which showed that both groups were motivated by the access to medication and study facilitators such as transportation and incentives. One potential reason for dropout regardless of the positive trial experience for some participants was due to the study helping them meet their desired goal of lowering BP and no longer needing supports. The higher average scores among non-completers with study staff satisfaction demonstrate no direct attribution to study staff as reasons for dropout.
Reasons for leaving the study	Participants who did not complete the study trial rated the following items as *very important* regarding reasons for leaving the study: possible risks to overall health (44%) and possibility of missing too much time at work (41%). Participants rated the following items as *neither important or unimportant*: possibility of adverse effects (44%) and possibility of making private medical information public (44%).	*Facilitators to recruitment and retention with proposed actionable next steps* Participants in the completer group also suggested the idea of hosting seminars highlighting participant experiences with high BP.	A prevalent reason for not completing the study was the concern around missing time from work. Participants in both groups noted the length of surveys and its influence on time, and made several recommendations to shorten the surveys, particularly questions that seemed repetitive. Doing so may help to lower participants’ concerns with missing too much time from work.

Participants’ experiences with the MI-BP trial were generally positive and consistent across completers and non-completers. One of the main reasons participants remained engaged throughout the study was their desire to monitor and maintain healthy BP levels and improve overall health. Participants accomplished this by accessing prescriptions throughout the study. Additionally, study facilitators such as transportation and monetary incentives, and a friendly study staff demonstrated to be effective in keeping participants engaged. Nevertheless, participants who dropped out reported meeting their goal of lowering BP. Others dropped out due to medication side effects or concerns about missing time from work. Overall, the MI-BP study had a positive effect on participants, with most favorably rating their satisfaction with the staff regardless of group.

## Discussion

The reasons for participation across both groups were influenced by both extrinsic and intrinsic motivations, such as access to healthcare, accessing BP cuffs, and learning about ways to control BP. These findings are consistent with research investigating facilitators to recruitment, indicating that several factors, such as personal access to knowledge and resources, are the most critical to participation in clinical trials among Black individuals [24]. Notably, some participants stressed the importance of learning about BP among their community, highlighting the importance for culturally-tailored interventions, such as the MI-BP trial. Culturally-tailored interventions can improve health access, disease knowledge, and clinical outcomes across various communities [25]. We also found that participants in both groups were satisfied with the MI-BP study staff. One reason for this could be that participants in both groups may have equally built rapport and trust with study staff earlier in the study. George et al [26] found that trust in the researcher and the reputation of the research institution are common facilitators for engagement in a study among Black individuals.

The reasons for participant dropout varied along a continuum. For some participants, decreased BP was an indicator of meeting the study objectives, thus influencing their decision to drop out. This finding contradicts the common belief that participants tend to dropout of studies due to lack of success and augments their motivation to improve their health. In fact, regardless of study goals, some participants did not find it necessary to continue engaging in the study because they met their personal goal of lowering their BP. Nevertheless, it is critical to emphasize that even when participants are seeing positive outcomes from participation in a study, that they remain engaged throughout the completion of the study as this may bias results and undermine the trial’s perceived utility. One of the novel contributions of this study is highlighting the role of participant motivation in clinical trials and its relationship to dropout rates. Several studies have identified facilitators to participation in clinical trials including but not limited to compensation, altruism, learning about a disease and ways to make improvements to their health [27]. However, it is unknown if other clinical trials have identified one of the reasons for participant dropout in relation to a positive outcome. In this study, some participants who saw improvements in their BP dropped out of the study because they met their health goal. Clinicals trials should investigate reasons for dropout as we may uncover nuanced reasons related to positive health outcomes from participation in the clinical trial.

Only a few participants mentioned that COVID interrupted with their participation in the clinical trial. To increase retention due to COVID, the study team adapted the study protocol and moved data collection electronically. Nonetheless, this was a challenging time for all, and constant changes were occurring in society. Other participants chose to dropout due to adverse effects from the medication they were taking. Study facilitators including transportation, access to prescriptions, and monetary incentives were beneficial and contributed to increased retention rates among Black individuals in the MI-BP trial. However, findings show mixed conclusions on using incentives and healthcare access, particularly among Blacks individuals [4,26,29]. A higher incentive may be commensurate to the time participants spend on a study. Using community-engaged approaches with this population may be beneficial in establishing an adequate incentive before the onset of a clinical trial by understanding the needs and wants of the population to better tailor incentives. This may help enhance both recruitment and retention efforts.

Participants in the MI-BP trial mentioned additional reasons for dropout, such as the possibility of adverse effects, missing too much work time, and the risk of having private medical information made public. Specifically, participants in the non-completer group were more worried about length of study time and surveys than completers. These findings are consistent with existing literature as a common concern across minorities, specifically Black individuals, is the demand of needing to work multiple jobs, being a primary caretaker, and justifying the benefits of participating in a study with a perceived high risk for a disease [26,28]. This can also lead to unintentional outcomes from the treatment received in the clinical trial [26]. These findings hold critical implications for conducting clinical trials, how research teams address participants’ concerns about sensitive health information, and strategies to reduce study completion time.

Study findings reinforce the importance of a well-established consent process. As such, we present several recommendations to enhance the consent process in clinical trials. During the consent process, researchers traditionally disclose all relevant information about the study, including benefits and risks to participants, to make an informed decision on participation. However, equally important, participants must *understand* what they consent to [29]. One way to ensure both criteria are met is to encourage research teams to use teach-backs where study staff assess a potential participant’s understanding of the informed consent, including the study purpose, procedures, benefits, risks, and ability to withdraw [30]. This also allows research staff to offer participants study clarifications in real-time [30]. Conversations about data security and privacy should also occur during teach-backs. Up-to-date training on data security and safety should be encouraged among study teams to incorporate the most up-to-date procedures in safeguarding participant data.

### Limitations

The time between recruitment and data collection could have influenced participant recall of the trial. Most participants were screened as early as June 2019; however, data collection for the current study occurred in May 2023. Therefore, some participants may have limited recall of the MI-BP trial, affecting their ability to report their experiences fully. The COVID-19 pandemic itself was another limitation that potentially affected participation and retention. While efforts were made to shift to virtual modalities for follow-up, the challenges of the pandemic undoubtedly affected completion rates. Finally, we collected demographic data during baseline of the parent trial. Some participants in the non-completer group were consented but did not show up for their baseline appointment and completed the survey at a later time. Therefore, demographic data for some participants was not collected. The demographic data would have helped provide further insight into the sample composition and would have resulted in no missing data.

## Conclusion

Participant dropout rates in mHealth trials have typically been high, especially among Black participants [13,31] Nonetheless, this is one of the few studies exploring barriers and facilitators to participant recruitment and retention in an mHealth trial on lowering BP. The findings from this study provide insights to enhance the research process further when recruiting and retaining Black individuals in clinical trials and offer practical recommendations for study teams. Given the high participant dropout rates in mHealth trials, continued research is needed to explore the most effective participant recruitment and retention strategies, especially among ethnic and racial minorities, to increase their participation in research and make appropriate trial adaptations.

## Supporting information

10.1017/cts.2026.10738.sm001 Perez et al. supplementary material 1 Perez et al. supplementary material

10.1017/cts.2026.10738.sm002 Perez et al. supplementary material 2 Perez et al. supplementary material

10.1017/cts.2026.10738.sm003 Perez et al. supplementary material 3 Perez et al. supplementary material

## Supplementary material

The supplementary material for this article can be found at https://doi.org/10.1017/cts.2026.10738.

## Table 1. Long description

The table presents demographic data of fifty-two survey participants, categorized by education, insurance, employment, and annual income. It consists of four main categories with subcategories and corresponding percentages. Under education, 35.9% have lower-than-college education, 28.2% have some college education, and 35.9% have a college or technical degree. For insurance, 38.5% are on Medicaid, 28.2% have private health insurance, 18% are on Medicare, and 15.4% are uninsured. In terms of employment, 66.7% are employed, and 33.3% fall under other categories. Annual income is divided into less than ten thousand dollars at 30.8%, ten thousand to twenty-four thousand nine hundred ninety-nine dollars at 12.8%, twenty-five thousand to forty-nine thousand nine hundred ninety-nine dollars at 23.1%, fifty thousand dollars or more at 10.3%, and did not report at 23.1%. The table provides a detailed breakdown of the participants’ demographic characteristics.

Navigate back to Table 1..

## Table 2. Long description

The table compares reasons for joining a study between completers and non-completers, with median scores for various reasons. The reasons include advancing science, improving lives, personal health benefits, treatment options, monetary compensation, understanding the program, receiving more care, having something to do, and someone to talk with. The table has 10 rows and 5 columns, including columns for completers, non-completers, and p-values. Notable differences include monetary compensation, where completers had a median score of 3.0 and non-completers had a median score of 4.0.

Navigate back to Table 2..

## Table 3. Long description

A table with three columns and two rows detailing subthemes, summaries, and exemplar quotes related to motivations for participating in a blood pressure study. The subthemes include ‘Learning to improve health’ and ‘External motivators’, with summaries explaining personal health-related reasons and external stimuli as motivations. Exemplar quotes from participants highlight their desire to control blood pressure and the benefits of receiving study-related equipment.

Navigate back to Table 3..

## Table 4. Long description

The table presents data on participants’ reasons for continuing, stopping, and the influence of COVID-19 on their study participation. It is organized into three main subthemes: reasons for continuing the study, reasons for stopping the study, and the influence of COVID-19 on study participation. Each subtheme includes a summary and exemplar quotes from participants. The ‘Reasons for continuing study’ subtheme highlights factors such as monitoring blood pressure, desire to follow through, and several other reasons. The ‘Reasons for stopping study’ subtheme notes reasons like achieving the goal of lowering blood pressure, feeling better, and one participant not remembering the reasons. The ‘Influence of COVID-19 on study participation’ subtheme discusses changes to study participation due to the pandemic. The table has three columns: Subthemes, Summary, and Exemplar quotes, with each row providing detailed information under these headers.

Navigate back to Table 4..

## Table 5. Long description

A table with four columns and four rows detailing facilitators to recruitment and retention, including considerations, summaries, exemplar quotes, and actionable next steps. The table highlights barriers such as the length of surveys and facilitators like increased monetary incentives and hosting community seminars. Each row provides a summary of participant feedback, exemplar quotes, and proposed actionable steps to improve retention efforts.

Navigate back to Table 5..

## Table 6. Long description

The table presents a joint display of integrated quantitative and qualitative findings with corresponding meta-inferences. It compares reasons for joining the MI-BP study, experiences with the study, and reasons for leaving the study between completers and non-completers. The table has four rows and three columns. The columns are labeled Quantitative summary results, Qualitative themes and summary results, and Meta-inferences. The rows are labeled Reasons for joining the MI-BP trial study, Experiences with MI-BP trial, and Reasons for leaving the study. The table shows that participants’ reasons for joining the MI-BP study did not differ significantly between completers and non-completers. Both groups were motivated by a desire to learn how to improve their blood pressure and by external factors such as receiving a blood pressure cuff or Fitbit. The table also highlights that both groups experienced positive aspects of the study, with completers finding the consent form easier to understand and the study length appropriate. Non-completers often left due to concerns about missing time from work or overall health risks. The table provides a detailed comparison of participant experiences and motivations, offering insights into the factors that influence study completion.

Navigate back to Table 6..
