# Supplementary material for: Barriers and facilitators to participant recruitment and retention among black adults in a mobile health intervention to control hypertension (MI-BP): A mixed methods study
Source: J Clin Transl Sci. 2026 May 6;10(1):e95. doi: 10.1017/cts.2026.10738 (PMC13247782; doi:10.1017/cts.2026.10738)
Supplement: Perez et al. supplementary material 1 — Perez et al. supplementary material [file S2059866126107389sup001.docx]

**Supplementary Appendix**

| **GRAMMS Reporting Guidelines** | **Reported on Page #** |
| --- | --- |
| (1) Describe the justification for using a mixed methods approach to the research question | Page 7; last paragraph in Introduction |
| (2) Describe the design in terms of the purpose, priority and sequence of methods | Page 7-8; Under ‘Design’ section |
| (3) Describe each method in terms of sampling, data collection and analysis | Pages 7-12 |
| (4) Describe where integration has occurred, how it has occurred and who has participated in it | Page 12; Last two sentences in ‘Methods’ section and joint display |
| (5) Describe any limitation of one method associated with the present of the other method | Pages 20-21 |
| (6) Describe any insights gained from mixing or integrating methods | Pages 16-17 |
