# Supplementary material for: Barriers and facilitators to participant recruitment and retention among black adults in a mobile health intervention to control hypertension (MI-BP): A mixed methods study
Source: J Clin Transl Sci. 2026 May 6;10(1):e95. doi: 10.1017/cts.2026.10738 (PMC13247782; doi:10.1017/cts.2026.10738)
Supplement: Perez et al. supplementary material 2 — Perez et al. supplementary material [file S2059866126107389sup002.docx]

You previously enrolled in the MI-BP research study and we are doing brief surveys/interviews about participant experiences. Survey completers will receive $20, and those who complete early will enter a drawing to win an additional $50-$100.

Interview completers will receive $100. To participate go to LINKGOESHERE.

**Group A: Completers**

**Why did you join?**

People have different reasons for signing up for a study like this. We’d like to find out why you joined the MI-BP study and how important these reasons are to you. (5-point scale 5=very important; 1=not at all important)

Help advance science and the treatment of high blood pressure Help save or improve the lives of other patients

Help improve my own high blood pressure Represent the best treatment option

the monetary compensation provided for participation

Guide understanding of how well a new blood pressure program works Receive more care and attention from health professionals

Give me something to do Give me someone to talk with

Other reasons (please list):

Source: Anderson et al. (2018)

# Do you feel like you were active in your participation?

Yes No Unsure

# [DISPLAY IF response is No or Unsure] Why did you not participate more?

We are interested to know if any of these reasons influenced how much you participated in the study. We would like to know how important these reasons were to you. (5-point scale 5=very important; 1=not at all important)

Possibility of adverse effects Possible risks to my overall health

[DISPLAY IF randomized to Control] Unhappy or not as interested as I did not receive the intervention/cell phone app

Possibility of making my private medical information public Possibility of missing too much time at work

Other reasons (please list):

# Experiences with MI-BP

Please reflect on your experience with the MI-BP study as a whole and rate your agreement with the following: (Likert-type 1-5)

The consent form was easy to understand The study fit well into to my daily routine The number of study visits was just right The length of study visits was just right

The overall amount of time the study took was about right Receiving compensation was convenient

The compensation was enough

# Satisfaction with MI-BP Staff

We are also interested in your experience with study staff. Please reflect on the study staff and rate your agreement with the following: (Likert-type 1-5)

Study staff were helpful in deciding whether to participate Study staff were helpful when I had questions

Study staff communicated with me at times that worked for me

Study staff communicated with me in my preferred method (e.g., text, call, etc) [DISPLAY IF assigned to the Intervention]: Study staff were good at orienting me

to the app/intervention

# Satisfaction with MI-BP Intervention

Please reflect on your experience with the intervention material and rate your agreement with the following: (Likert-type 1-5)

The educational material was helpful to manage my blood pressure The blood pressure cuff was easy to use

[DISPLAY IF assigned to the Intervention]: The medication reminders were helpful

I understood what my medication dosage should be

[DISPLAY IF assigned to the Intervention]: Overall, the MI-BP intervention help

me to manage my blood pressure

[DISPLAY IF assigned to the Intervention]: The app was easy to use [DISPLAY IF assigned to the Intervention]: The Fitbit was helpful to me to be physically active

Based on your experience, would you volunteer for MI-BP if you had it to do over again?

Yes

No

How likely would you be to recommend MI-BP or a similar program to a good friend or family member if they were eligible? (10-point Likert-type)

What would have made your experience with MI-BP better? [open-ended]

Would you be willing to be contacted to participate in a 15-30 minute interview about your experiences with MI-BP?

Yes No

**Thank you very much for participating in this survey.**

**Group B: Non-Completers**

**Why did you join?**

People have different reasons for signing up for a study like this. We’d like to find out why you joined the MI-BP study and how important these reasons are to you. (5-point scale 5=very important; 1=not at all important)

Help advance science and the treatment of high blood pressure Help save or improve the lives of other patients
Help improve my own high blood pressure Represent the best treatment option

the monetary compensation provided for participation

Guide understanding of how well a new blood pressure program works Receive more care and attention from health professionals

Give me something to do Give me someone to talk with

Other reasons (please list):

Source: Anderson et al. (2018)

# Experiences with MI-BP

Please reflect on your experience with the MI-BP study as a whole and rate your agreement with the following: (Likert-type 1-5)

The consent form was easy to understand The study fit well into to my daily routine The number of study visits was just right The length of study visits was just right

Based on your experience, would you volunteer for MI-BP if you had it to do over again?

Yes No

# **Why did you leave the study?

People also have different reasons for leaving a study. We would like to know how important these reasons were to you. (5-point scale 5=very important; 1=not at all important)

Possibility of adverse effects Possible risks to my overall health

[DISPLAY IF not randomized] Possibility of not receiving the intervention/cell phone app

[DISPLAY IF randomized to Control] Unhappy or not as interested as I did not receive the intervention/cell phone app

Possibility of making my private medical information public Possibility of missing too much time at work

Other reasons (please list):

***What would have the MI-BP study more appealing to you? [open-ended]

Would you be willing to be contacted to participate in a 15-minute interview about your experiences with MI-BP?

Yes

No

**Thank you very much for participating in this survey.**
