# Supplementary material for: Barriers and facilitators to participant recruitment and retention among black adults in a mobile health intervention to control hypertension (MI-BP): A mixed methods study
Source: J Clin Transl Sci. 2026 May 6;10(1):e95. doi: 10.1017/cts.2026.10738 (PMC13247782; doi:10.1017/cts.2026.10738)
Supplement: Perez et al. supplementary material 3 — Perez et al. supplementary material [file S2059866126107389sup003.docx]

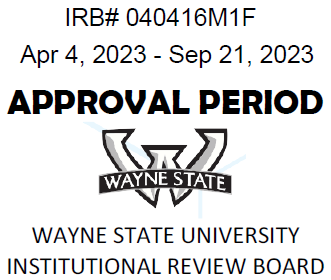
**MI-BP Experience: Patient Interview Guide**

# [NOTE: THIS DOCUMENT IS TO BE TREATED AS A GUIDE, NOT A SCRIPT. THE INTERVIEWER MAY DEVIATE FROM THE WRITTEN TEXT TO CLARIFY RESPONSES, FURTHER EXPLORE A TOPIC, AND TO ENSURE ADEQUATE INFORMATION IS OBTAINED]

| **MI-BP Trial Experience** | | |
| --- | --- | --- |
| Interviewer: | | |
| Interviewee Code: | | |
| Date: | Time: | Place: Zoom |
| Interviewer Introduction (read aloud) | | |
| As part of the *MI-BP: mHealth to Improve Blood Pressure Control* Project (PI: Levy) we are interviewing participants in the study to further understand barriers and facilitators to success with the MI-BP intervention and their experiences with the trial.  You can skip any questions during this interview that you don’t want to answer, whatever the reason, and you don’t have to tell us why. You may also refuse to have the interview session audio recorded.  To keep your information confidential, we will store all study data in secure and access-limited folders. The audio files will be destroyed after a transcript has been made, and the transcript will not identify you by name.  In return for your participation, you will receive $XX via ClinCard. | | |
| Do you have any questions before we begin? Y/N | | |
| Do you consent to participate in this interview? Y/N | | |
| May I start recording the session? Y/N | | |
| Interviewer states: “This is [staff member], and I’m with participant [code]. Do I have your permission to record this interview?” Y/N  *Do not proceed until the participant has confirmed.* | | |

**Interview Questions/Prompts Initial Experience:**

*First, I have some broad questions about your initial experience with the MI-BP study.*

1. If you recall, how did you first learn about the MI-BP study?
2. What are some reasons why you agreed to participate in the MI-BP study?

3. What interested you about the study initially?

Possible prompts

- - - Money? Study swag?
    - Bored waiting in the ED?
    - Your health?
    - Improve other’s health?
    - Others?

4. What were your thoughts about the study initially? How well did you trust the study staff?

1. Interested?
2. Committed?

5. Do you recall when you first learned about the study (e.g., emergency department visit or community event)? How well did you trust the volunteers who explained the study to you?

6. Had you participated in a research study before? If so, how did that go?

# Patient engagement:

*Now, I’d like to switch gears a bit to ask about your experience after you enrolled in the study.*

7. Do you remember the blood pressure cuff, Fitbit, and mobile app? If so, what did you think of it?

1. BP cuff?
2. Fitbit?
3. App?

8. [INTERVENTION GROUP ONLY] How helpful was the app in managing your blood pressure?

9. Study staff tried to contact participants for reminders and next steps. We are wondering if these calls went through, if you remember them, and whether they were helpful?

10. Did you have any concerns about confidentiality or privacy? If so, can you please explain or give an example.

11. What did you think about the study visits?

1. How convenient were study visits?
2. What were your thoughts about in person visits versus a virtual visit?
3. How was your interaction with study staff?
4. What did you think of follow-up with the pharmacist?

12. What did you think about the amount your time and effort in participating in the study?

1. Tracking blood pressure
2. Tracking physical activity

13. What are some reasons that you stayed in the MI-BP study?

1. Money? Study swag?
2. Your health?
3. Helped improve your blood pressure?
4. Improve other’s health?
5. Others?

14. [INTERVENTION GROUP ONLY] Thinking about the app, what might have helped you use it even more than you did?

15. What kept you from participating more in the study?

a. What could have been done to help and make it better?

16. What would have made the study more attractive or appealing to participate in?

a. Thoughts on the incentive (enough amount and the use of ClinCard)

17. [FOR PARTICIPANTS DURING COVID]. How did participating in the study look for you during COVID as compared to before COVID?

1. Time spent participating
2. Study visits
3. Other concerns

# Wrap up:

18. I appreciate your answer to all of our questions. What else might be important for us to know that we have not asked about?

*Thank you very much for your time.*
